# Vaccination with immune complexes modulates the elicitation of functional antibodies against HIV-1

**DOI:** 10.3389/fimmu.2023.1271686

**Published:** 2023-10-03

**Authors:** Catarina E. Hioe, Xiaomei Liu, Andrew N. Banin, Daniel W. Heindel, Jéromine Klingler, Priyanka G. Rao, Christina C. Luo, Xunqing Jiang, Shilpi Pandey, Tracy Ordonez, Philip Barnette, Maxim Totrov, Jiang Zhu, Arthur Nádas, Susan Zolla-Pazner, Chitra Upadhyay, Xiaoying Shen, Xiang-Peng Kong, Ann J. Hessell

**Affiliations:** ^1^ Division of Infectious Diseases, Department of Medicine, Icahn School of Medicine at Mount Sinai, New York, NY, United States; ^2^ Research Service, James J. Peters VA Medical Center, Bronx, NY, United States; ^3^ Department of Biochemistry and Molecular Pharmacology, New York University Grossman School of Medicine, New York, NY, United States; ^4^ Division of Pathobiology and Immunology, Oregon National Primate Research Center, Oregon Health and Science University, Beaverton, OR, United States; ^5^ Molsoft L.L.C., San Diego, CA, United States; ^6^ Department of Integrative Structural and Computational Biology and Department of Immunology and Microbiology, The Scripps Research Institute, La Jolla, CA, United States; ^7^ Department of Environment Medicine, New York University Grossman School of Medicine, New York, NY, United States; ^8^ Division of Surgical Sciences, Department of Surgery, Duke University School of Medicine, Durham, NC, United States

**Keywords:** HIV-1 vaccine, HIV-1 Env, antibody, immune complex (IC), virus neutralization, ADCP

## Abstract

**Introduction:**

Neutralizing antibodies (Abs) are one of the immune components required to protect against viral infections. However, developing vaccines capable of eliciting neutralizing Abs effective against a broad array of HIV-1 isolates has been an arduous challenge.

**Objective:**

This study sought to test vaccines aimed to induce Abs against neutralizing epitopes at the V1V2 apex of HIV-1 envelope (Env).

**Methods:**

Four groups of rabbits received a DNA vaccine expressing the V1V2 domain of the CRF01_AE A244 strain on a trimeric 2J9C scaffold (V1V2-2J9C) along with a protein vaccine consisting of an uncleaved prefusion-optimized A244 Env trimer with V3 truncation (UFO-BG.ΔV3) or a V1V2-2J9C protein and their respective immune complexes (ICs). These IC vaccines were made using 2158, a V1V2-specific monoclonal Ab (mAb), which binds the V2i epitope in the underbelly region of V1V2 while allosterically promoting the binding of broadly neutralizing mAb PG9 to its V2 apex epitope *in vitro*.

**Results:**

Rabbit groups immunized with the DNA vaccine and uncomplexed or complexed UFO-BG.ΔV3 proteins (DNA/UFO-UC or IC) displayed similar profiles of Env- and V1V2-binding Abs but differed from the rabbits receiving the DNA vaccine and uncomplexed or complexed V1V2-2J9C proteins (DNA/V1V2-UC or IC), which generated more cross-reactive V1V2 Abs without detectable binding to gp120 or gp140 Env. Notably, the DNA/UFO-UC vaccine elicited neutralizing Abs against some heterologous tier 1 and tier 2 viruses from different clades, albeit at low titers and only in a fraction of animals, whereas the DNA/V1V2-UC or IC vaccines did not. In comparison with the DNA/UFO-UC group, the DNA/UFO-IC group showed a trend of higher neutralization against TH023.6 and a greater potency of V1V2-specific Ab-dependent cellular phagocytosis (ADCP) but failed to neutralize heterologous viruses.

**Conclusion:**

These data demonstrate the capacity of V1V2-2J9C-encoding DNA vaccine in combination with UFO-BG.ΔV3, but not V1V2-2J9C, protein vaccines, to elicit homologous and heterologous neutralizing activities in rabbits. The elicitation of neutralizing and ADCP activities was modulated by delivery of UFO-BG.ΔV3 complexed with V2i mAb 2158.

## Introduction

Developing HIV envelope (Env) immunogens capable of eliciting antibodies (Abs) effective against a broad array of HIV-1 isolates is a major challenge in HIV vaccine development. Phase 2b/3 vaccine trials, including the recent HVTN 706, HVTN 705, and HVTN 702 trials testing different vaccine platforms and regimens to elicit cross-reactive Abs against Env, have yielded no efficacy signals (1–3). HIV-1 vaccine candidates designed to generate broadly neutralizing Abs (bNAbs) have not attained their ultimate goals, although a germline-targeting strategy utilizing a self-assembling nanoparticle vaccine with 60 copies of gp120 engineered outer domain (eOD-GT8 60mer) was reported to stimulate precursors of VRC01-class bNAbs against the CD4-binding site (CD4bs) in nearly all vaccine recipients in a phase I trial (4). During HIV-1 infection, bNAbs are produced after multiple years of chronic infection only in a small subset of HIV-1–seropositive individuals (5–8). Although exposure to diverse variants over years has been implicated in promoting or guiding bNAb development and maturation (9, 10), other factors contributing to the generation of bNAbs are not fully understood.

This study sought to examine whether anti-Env Abs that form immune complexes (ICs) could exert any modulatory effects on Ab responses against bNAb epitopes on Env. Gach et al. utilized IC vaccines to suppress Ab responses to the immunogenic, strain-specific glycan hole on the BG505 SOSIP.664 trimer, but the blockage did not divert the Ab responses toward broadly reactive neutralizing epitopes (11). Other studies tested IC vaccines of the gp120 core cross-linked or fused with CD4i mAbs against the bridging sheet that preferentially expose the CD4bs epitope for the VRC01 bnAb lineage. Immunization with these ICs promoted the generation of Abs with similar binding footprints as the VRC01-class bNAbs (12, 13). A similar study examined IC vaccines of gp120 cross-linked with mAb A32 to allosterically stabilize the chemokine receptor-binding site, but IC-induced neutralizing titers were comparable to those attained by gp120 alone (14). In earlier studies, we also observed the allosteric effects of CD4bs mAbs that enhanced exposure and stability of the crown region of the V3 loop on gp120, resulting in greater Ab reactivity against V3 (15–18). Immunization with ICs made of gp120 and a CD4bs mAb elicited higher levels of cross-reactive Ab responses against the V3 crown, but the neutralizing activity was limited to tier 1 viruses and ineffective against most HIV-1 strains that express Env with an occluded V3 crown (16).

Allosteric effects were similarly observed within the V1V2 domain of Env upon binding by certain V1V2-specific mAbs. In the Env trimer, three V1V2 domains create the apical cap, and each V1V2 domain is a modular 5-strand beta-barrel that includes a V2C strand capable of adopting polymorphic structures (19). The V2C peptide is recognized by V2p class mAbs, such as CH58, CH59, and CAP228.3D, in alpha-helical configurations (20). A second class of mAbs targets the V2-apex (aka V2 glycan, V2 quaternary, or V2q) that includes the prototypic bNAbs PG9, PG16, PGDM1400, CH01, and PGT145. These V2-apex bNAbs preferentially bind Env as trimers, recognize *N*-glycans on the V1V2 apical surface as part of their epitopes, and require that the V2C-strand assumes a beta-sheet conformation (21–25). The V2w class mAbs from rhesus macaques infected with chimeric simian-chimpanzee immunodeficiency viruses were recently reported and shown to target the V2 apex, but these mAbs neutralize weakly because the V2w epitopes are occluded in the native closed Env conformation (26). The fourth class of mAbs, V2i, targets epitopes near the integrin α4β7-binding motif at the underbelly of V1V2 (19). Interestingly, the binding of V2i mAbs 2158 and 697 was observed to augment V1V2 recognition by PG9 or CH01, respectively (15), indicating the capacity of V2i mAbs to induce allosteric changes favoring the beta-sheet structure required for the V2 apex bNAb binding.

In our past study, immunization of mice with IC vaccines composed of monomeric gp120 proteins and V2i mAb 2158 yielded either a higher V2 Ab response with no neutralizing activities or an enhanced V3 Ab response with only tier 1 virus neutralization (15). The present study sought to investigate the effect of ICs on the elicitation of neutralizing Ab responses against the V2 apex on the Env trimer. To this end, an uncleaved prefusion-optimized (UFO) gp140 Env trimer of CRF01_AE A244 with V3 truncation (UFO-BG.ΔV3) and A244 V1V2 on a trimeric 2J9C scaffold (V1V2-2J9C) were examined as uncomplexed proteins (UC) or in complex with mAb 2158 (IC) for immunogenicity in rabbits. A V1V2-2J9C DNA vaccine previously shown to promote the targeting of Ab responses to the V2 apex (27) was also incorporated for a co-immunization regimen. The data demonstrated that V1V2-specific Abs with distinct binding profiles were produced in the groups immunized with DNA and UFO-BG.ΔV3 UC or IC vaccines versus DNA and V1V2-2J9C UC and IC vaccines. Limited heterologous neutralizing activities were detected only in the DNA/UFO UC group. Immunization with DNA/UFO IC caused varying degrees of alterations in the induction of V1V2-binding Abs, Abs that neutralize autologous tier 1 virus via the N160-glycan–dependent V2-apex epitope, and V1V2-specific Abs that mediate phagocytosis. Thus, these data demonstrate the modulatory effects of ICs on the generation of functional Abs against HIV-1.

## Materials and methods

### Rabbit immunization

Rabbit immunization studies were performed in the Center for Comparative Medicine and Surgery (CCMS) in the Icahn School of Medicine at Mount Sinai (ISMMS). The ISMMS CCMS is accredited by the American Association for the Accreditation of Laboratory Animal Care International and adheres to the Guide for the Care and Use of Laboratory Animals and the U.S. Public Health Service Policy on the Humane Care and Use of Laboratory Animals. Animal care and experimentation were performed in accordance to a protocol approved by the ISMMS Institutional Animal Care and Use Committee.

Rabbits were co-immunized with V1V2-2J9C DNA plasmid with a gene gun [Particle Mediated Epidermal Delivery (PMED) device, XR-1 research model, Oxford, UK] and one of the protein immunogens (UFO-BG.ΔV3 or V1V2-2J9C) as uncomplexed vaccines (UC) or IC vaccines with V2i mAb 2158. Each rabbit received four doses of DNA and protein vaccines at 4-week intervals. Blood was collected before immunization was commenced and 2 weeks after each vaccination.

The plasmid 418H encoding A244 V1V2-2J9C with tissue plasminogen activator (tPA) signal sequence under HCMV promotor was used as a DNA vaccine for all four groups of rabbits and was a gift from Dr. Barbara Felber (27). In preparation for gene gun delivery, the DNA plasmid was precipitated onto 1-μm-diameter gold beads (2 μg of DNA/mg of gold), and cartridges carrying the DNA (“bullets”) were prepared according to the manufacturer’s instructions. To verify the functional expression, COS-7 cells were transfected with DNA carried by the gold beads. Briefly, COS-7 cells (2 × 10^5^ cells per chamber) were seeded in two-chamber culture slides (Thermo Scientific Nunc) and transfected the next day by a gene gun at a pressure of 125 lb/in^2^. Cells were incubated at 37°C for 2–3 days and were fixed and labeled with mAb (2158 or PG9; 6 μg/mL) and a 1:100 dilution of fluorescein isothiocyanate (FITC)–conjugated goat anti-human IgG (Zymax, Invitrogen). The presence of transfected cells was revealed by FITC staining visualized by fluorescence microscopy.

The UFO-BG.ΔV3 gp140 protein was constructed with an A244 gp120 subunit, a gp41 ectodomain of BG505, and an optimized helical region 1 to form an uncleaved chimeric Env trimer (28, 29). The V3 crown (RIGPGQA) was deleted, and two amino acids were substituted (R319Y and T320F according to HXB2 location). The goal of this modification was to eliminate major V3 crown immunogenic epitopes while minimizing impact on closed trimer stability. Our previous study of the mutation effects on neutralization sensitivity (30) indicated that the V3 crown could be released from its pocket on the trimer without overall trimer opening, suggesting that a well-designed truncation may achieve this goal. Models of the V3 loop with varying crown segments (starting at positions 306–309 and ending at 316–319) replaced by two-residue connector were built and reviewed (all modeling was performed in ICM-pro, Molsoft, San Diego). Models indicated that low-strain beta-turn could be formed at several lengths, but extensive truncations involving I307 and F317 left a large cavity that we anticipated might disrupt the Env apex. We therefore focused on the design that replaced segment 308:316. A connector residue pair …GR… was selected on the basis of observed residue frequencies in structurally similar loops (loop preferred residue tool, also in ICM-pro). Further V3 stability optimization was attempted at buried but hydrophilic positions R319 and T320, where a computational mutation scan suggested Y and F substitutions. Notably, Y319 and F320 are also found in one of the optimized SOSIP trimer structures, Protein Data Bank (PDB) 6NFC (31). Truncated versions without and with R319Y/T320F substitutions were expressed and the latter construct with a slightly better antigenic profile was chosen.

The UFO-BG proteins with or without V3 alterations were produced in ExpiCHO-S cells using the ExpiFectamine CHO Transfection Kit (Gibco) and purified by lectin affinity chromatography (Galanthus nivalis lectin (GNL), Vector Laboratories) followed by gel filtration on a Superdex 200 column (GE). The V1V2-2J9C protein was produced in 293S (GnTI−/−) cells and purified by affinity chromatography as described (32). IC vaccines were prepared at an antigen:mAb ratio of 1:3 with V2i mAb 2158 and then mixed with adjuvants. UC vaccines were mixed at the same ratio with an irrelevant anti-parvovirus mAb 860. The protein vaccines were administered subcutaneously with MPLA (monophosphoryl–lipid A; Avanti, #699800P) and DDA (dimethyldioctadecylammonium bromide; Sigma, # D2779) adjuvants.

### ELISA

Enzyme-linked immunoassay (ELISA) was used to verify the antigenicity of immunogens used in the immunization experiments and characterize the mAb reactivity of antigens used in the multiple bead assays (33). In brief, antigens were coated on the ELISA wells and reacted with mAbs followed with an enzyme-coupled anti-human immunoglobulin G (IgG) secondary Ab. Irrelevant anti-parvovirus mAbs 1418 or 860 were included as negative controls. Colorimetric and luminescent substrates were used interchangeably, and the respective optical density or relative luminescence unit (RLU) readings were shown. To examine the allosteric effects of 2158 on PG9 binding, 2158-bound ICs were probed with biotinylated PG9, which was then detected with an alkaline phosphatase–conjugated streptavidin.

### Octet bio-layer interferometry

To evaluate the antigenic property of V1V2-2J9C expressed by plasmid 418H, the supernatant from 418H-transfected 293T cells was subjected to bio-layer interferometry (BLI) analysis using an Octet Red96 instrument (ForteBio) as previously described (34). V1V2-specific mAbs (5 µg/mL) were immobilized on anti-human immunoglobulin G fragment (hIgG Fc) capture (AHC) biosensors and reacted with serially diluted supernatant. Supernatant from non-transfected 293T cells was used as an assay buffer. The baseline response was established by running the loaded AHC sensors on a blank control and subtracted from the binding curves.

### Multiplex bead Ab-binding assay

To measure the relative levels of rabbit IgG reactive to different Env and V1V2 antigens, Luminex multiplex bead assays were performed with a panel of antigens described in (35). The following reagents were obtained through the National Institutes of Health (NIH) HIV Reagent Program, Division of Acquired Immunodeficiency syndrome (AIDS), National Institute of Allergy and Infectious Diseases (NIAID), NIH: recombinant proteins AE.A244 delta11 gp120 (ARP-12569), C.1086 gp140C (ARP-12581), C.1086 gp120 D7 (ARP-12582), M.CON-S delta11 gp120 (ARP-12576), C.1086 V1V2 tags, and AE.A244 V1V2 tags, contributed by Drs. Barton F. Haynes and Hua-Xin Liao. V1V2 on 1FD6, 2J9C, or 1KNC scaffolds were produced the labs of Drs. Hioe or Xiang-Peng Kong (32, 36). Cyclic V2 peptides were made commercially with an N-terminal 6x Lys-Gly. Antigens were coupled to beads with the xMAP Antibody Coupling (AbC) Kit (Luminex). Rabbit IgG binding to the bead mixture was performed as described (35), except that biotinylated mouse anti-rabbit IgG (SouthernBiotech) was used. For PG9- and 2158-blocking assays, beads coated with the specified antigens were treated with serially diluted rabbit sera and then reacted with biotinylated PG9 or 2158.

### Neutralization assay

Neutralizing activity of rabbit serum was evaluated using HIV-1 pseudoviruses in TZM.bl reporter cells as described previously (37, 38). Neutralization was measured as reduction of luciferase reporter gene expression after a single round of infection. This assay has been formally optimized and validated (39) and was performed in compliance with Good Clinical Laboratory Practices, including participation in a formal proficiency testing program (40). Viruses were selected from the global HIV-1 reference strains (41). In the standard neutralization assays, serially diluted rabbit serum was incubated with pre-titrated pseudovirus for 1 h and then added to TZM.bl cells. Neutralization titers [50% inhibitory dose (ID_50_)] were determined as the serum dilution at which RLUs were reduced by 50% compared to RLU in virus control wells after subtraction of background RLU in cell control wells.

For antigen-blocking neutralization assays, diluted rabbit serum was pre-treated with a fixed amount of antigen at the designated concentration, followed by neutralization assay that was performed as previously described (34). The mixture was incubated with pseudovirus for 1 h and then added to TZM.bl cells. TZM-bl cells (also called JC53-BL13) were obtained from the NIH AIDS Research and Reference Reagent Program, as contributed by John Kappes and Xiaoyun Wu.

### Antibody-dependent cellular phagocytosis assay

The ability of V1V2-specific Abs in rabbit serum to mediate Ab-dependent cellular phagocytosis (ADCP) was evaluated as previously described using fluorescent NeutrAvidin beads conjugated with A244 V1V2-2J9C and THP-1 phagocytic cells American Type Culture Collection (ATCC) (15, 34). Mean fluorescent intensity was measured, and ADCP scores over serum dilutions were calculated.

### Data analysis

The binding and functional activities of serially diluted Abs were calculated as areas under the titration curves (AUCs) or ID_50_ titers. These values were subtracted by background levels of the particular assays, and delta AUCs or ID_50_ values were depicted. Statistical analysis was performed using the designated statistical tests in GraphPad Prism or R software. Correlation matrices were generated using R version 4.1.0 (The R Foundation for Statistical Computing) and corrplot package.

## Results

### Combination of DNA and protein vaccines used to target Ab response to the V1V2 apex

**Figure 1:**
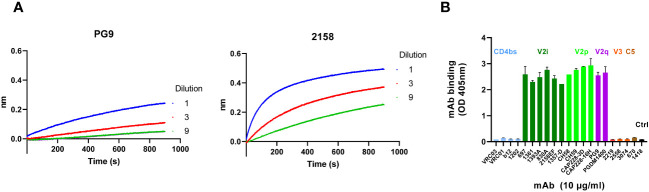
Antigenicity profile of V1V2-2J9C. **(A)** Culture supernatant from 293T cells transfected with the A244 V1V2-2J9C–encoding plasmid (418H) was tested using Octet bio-layer inteferometry (BLI) for reactivity with PG9 and 2158. Each mAb (5 µg/mL) was applied onto anti-human IgG Fc (AHC) biosensors and reacted with titrated amounts of V1V2-2J9C-containing supernatant. The V1V2-2J9C binding was measured over time. **(B)** ELISA reactivity of A244 V1V2-219C with mAbs against different Env regions. The V1V2-2J9C protein (2 µg/mL) was coated onto ELISA wells and reacted with mAbs (10 µg/mL each) specific for the CD4bs, V1V2 (V2i, V2p, and V2q), V3, C5, or irrelevant parvovirus antigen.

To direct Ab responses to the V1V2 apex of HIV-1 Env, a DNA + protein co-immunization strategy was employed. The V1V2-2J9C DNA vaccine expressing V1V2 of CRF01_AE A244 was delivered by a gene gun. This construct was selected because it expressed a V1V2-2J9C protein reactive with PG9 bnAb against the V2 apex (**Figure 1A**, left). The protein also reacted strongly with V2i mAb 2158 against the underbelly region of V1V2 (**Figure 1A**, right). A similar reactivity pattern was also apparent upon transfection of COS-7 cells with the DNA vaccine using gene gun (**Figure S1**). ELISA analysis further showed that V1V2-2J9C was recognized by human mAbs against the three known types of V1V2 epitopes: V2i, V2p, and V2q (**Figure 1B**).

**Figure 2:**
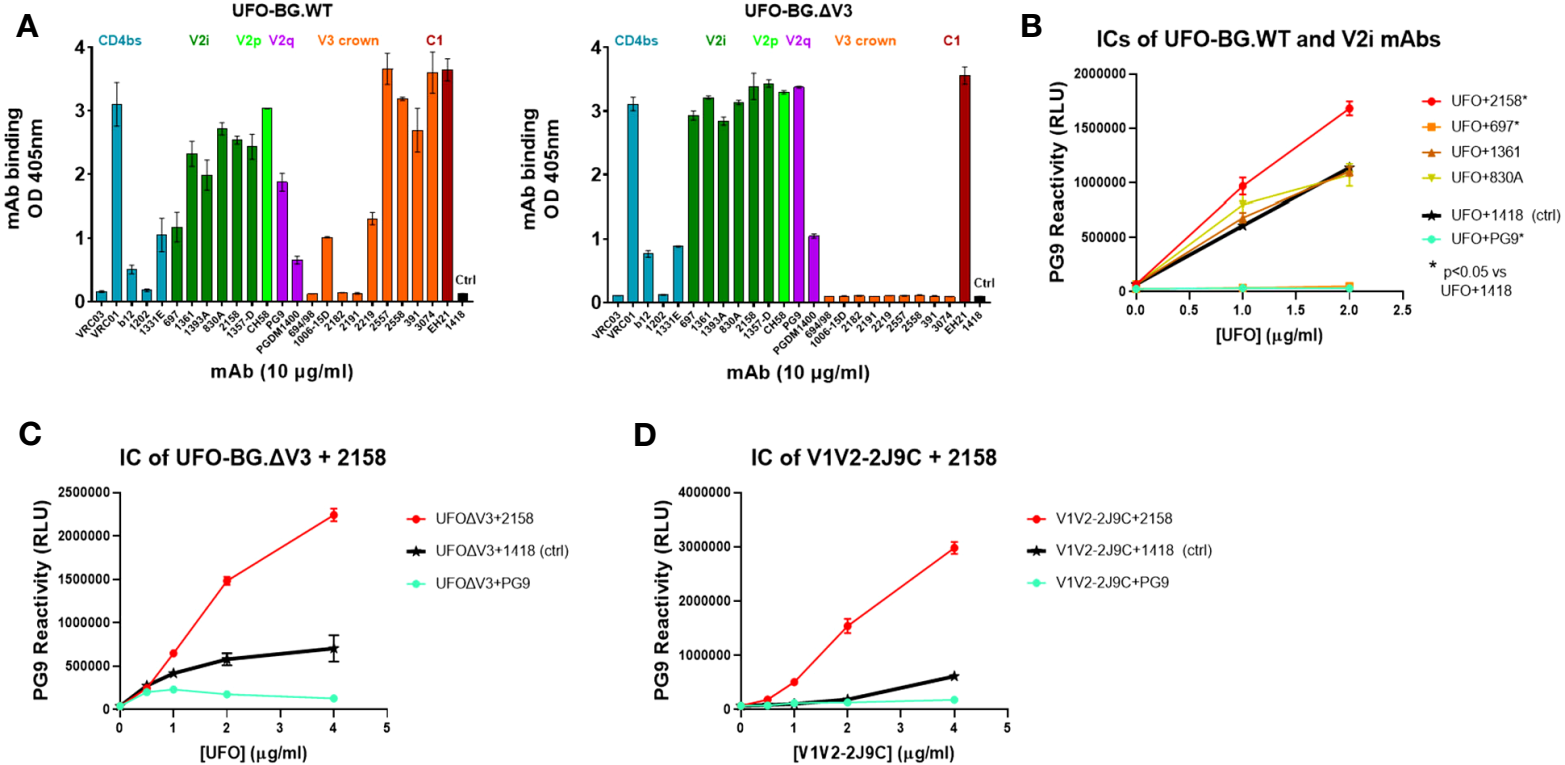
Antigenicity of different A244 UFO-BG Env and V1V2 constructs. **(A)** Reactivity of UFO-BG.WT vs. UFO-BG.ΔV3 with mAbs against different Env regions to verify the loss of anti-V3 mAb reactivity with UFO-BG.ΔV3. **(B)** Enhanced PG9 reactivity against UFO-BG.WT in complex with 2158 but not the other V2i mAbs. **(C)** Enhanced PG9 reactivity similarly observed with UFO-BG.ΔV3 in complex with 2158. **(D)** Enhanced PG9 reactivity also detected against V1V2-2J9C in complex with 2158. ELISA was performed by coating wells with antigens at 2 µg/mL or titrating amounts followed by incubation with the designated mAbs. In panel **(A)**, mAb binding was detected with an alkaline phosphatase (AP)–coupled secondary anti-human IgG antibody. For panels **(B–D)**, the different antigens were pre-incubated with each mAb at a 1:3 ratio. Uncomplexed antigens were treated an irrelevant anti-parvovirus mAb 1418 for controls. Respective antigens pre-treated with PG9 were tested in parallel. The antigen and mAb mixtures were diluted serially, coated onto wells, and reacted with biotinylated PG9, followed with AP-streptavidin and luminescent substrate.

To design the protein vaccine, we produced a trimeric UFO Env bearing A244 gp120 with a stabilized BG505 gp41 design (28, 29). The A244 UFO Env (UFO-BG.WT) was further modified by removing part of the V3 crown (UFO-BG.ΔV3) in order to direct Ab responses away from the immunogenic V3 crown epitopes. This deletion, as expected, abrogated the reactivity with V3 crown–specific mAbs, whereas the binding of mAbs against the different V1V2 epitopes, the CD4 binding site, and the N-terminal C1 region was comparable to that of UFO-BG.WT (**Figure 2A**). These findings indicate that the Env antigenic profiles outside the V3 crown were minimally altered.

In our previous study (15), an IC of A244 gp120 and V2i-specific mAb 2158 was found to display enhanced reactivity with the V2 apex-specific bnAb PG9. Here, we tested whether this allosteric effect could be observed with ICs made of UFO-BG.WT and UFO-BG.ΔV3 (**Figures 2B, C**). The data in **Figure 2B** show a greater PG9 reactivity with 2158-bound versus uncomplexed UFO-BG.WT treated with an irrelevant mAb 1418. The enhancing effect was specific for 2158; V2i mAbs 1361 and 830A did not change PG9 binding, whereas V2i mAb 697 inhibited PG9 binding, most likely due to steric hindrance. The enhanced PG9 reactivity was similarly observed on UFO-BG.ΔV3 in complex with 2158 (**Figure 2C**). This allosteric effect was also noticed when 2158 formed an IC with A244 V1V2-2J9C (**Figure 2D**) and other V1V2 scaffolds (ZM53 V1V2-2J9C or ZM233 V1V2-1FD6) (**Figure S2**). To target Ab responses toward the V1V2 apex, UFO-BG.ΔV3 and V1V2-2J9C of A244 were selected as protein immunogens for comparison with their respective ICs made with mAb 2158. To estimate the affinity of 2158 for UFO-BG.ΔV3 and V1V2-2J9C, the half maximal binding was determined by ELISA to yield 0.62 nM (95% confidence interval: 0.34-1.07 nM) and 0.15 nM (95% confidence interval: 0.14-0.17 nM), respectively.

### Rabbit immunization with DNA + protein combination

**Figure 3:**
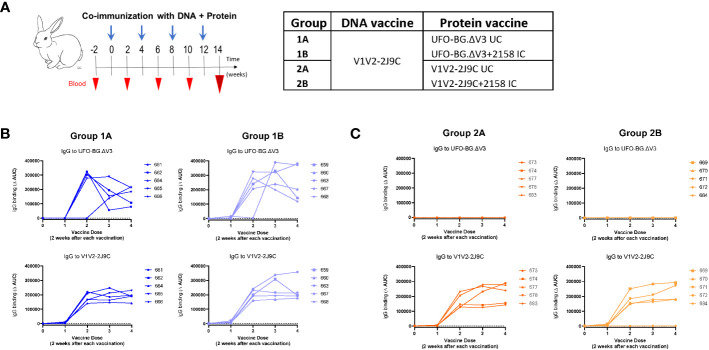
Immunization of rabbits with DNA and protein vaccines to elicit Abs responses to V1V2. **(A)** Rabbits (n = 5 per group) received V1V2-2J9C-encoding DNA vaccine by gene gun and a protein vaccine made of UFO-BG.ΔV3 or V1V2-2J9C, either uncomplexed (UC) or as immune complex (IC) with V2i mAb 2158. Each rabbit was immunized four times at 1-month intervals, and blood was collected 2 weeks after each vaccine dose. **(B)** Induction of Ab responses against the homologous UFO-BG.ΔV3 or V1V2-2J9C antigens over time in Group 1A I (DNA/UFO-UC) and Group 1B (DNA/UFO-IC). **(C)** Induction of Ab responses over time against V1V2-2J9C, but not UFO-BG.ΔV3, in Group 2A (DNA/V1V2-UC) and Group 2B (DNA-V1V2-IC). Rabbit ID numbers are denoted in panels **(B, C)**.

Four groups of rabbits (n = 5 per group) were tested for immunization with a DNA vaccine along with uncomplexed or IC protein vaccines. All rabbits were co-immunized with four doses of DNA and protein vaccines at 1-month intervals. The DNA vaccine (36 µg per dose) was delivered using a gene gun, and the protein vaccines were administered subcutaneously with MPLA (25 µg per dose) and DDA (250 µg per dose) adjuvants. For protein vaccines, antigen (100 µg per dose) and mAb (300 µg per dose) were mixed for 1 h before addition of adjuvants. Each rabbit received the same V1V2-2J9C DNA vaccine and one of the following protein vaccines: i) uncomplexed UFO-BG.ΔV3 (Group 1A = DNA/UFO-UC), ii) UFO-BG.ΔV3 + 2158 IC (Group 1B = DNA/UFO-IC), iii) uncomplexed V1V2-2J9C (Group 2A = DNA/V1V2-UC), and iv) V1V2-2J9C + 2158 IC (Group 2B = DNA/V1V2-IC) (**Figure 3A**). The uncomplexed protein vaccines were mixed with an irrelevant anti-parvovirus mAb 860. Blood was collected before immunization and 2 weeks after each vaccination to monitor the induction of serum IgG against UFO-BG.ΔV3 and V1V2-2J9C.

In Groups 1A and 1B (DNA/UFO-UC and IC), Abs against the homologous UFO-BG.ΔV3 were detected in all five rabbits after the third and fourth vaccinations (**Figure 3B**). Ab responses to V1V2 were also induced after the second vaccine dose and maintained with the subsequent doses. In Groups 2A and 2B (DNA/V1V2-UC and IC), high levels of Abs were generated against the homologous V1V2-2J9C starting after two vaccinations (**Figure 3C**). However, no to very low levels were detected against UFO-BG.ΔV3, indicating a poor recognition of V1V2 on UFO-BG.ΔV3 by the elicited Abs.

### Differential Ab responses induced by UFO-BG.ΔV3 vs. V1V2-2J9C proteins

To further examine the differential Ab responses induced in the four rabbit groups, serum specimens collected 2 weeks after the final immunization were tested for IgG reactivity against 15 antigens, including gp140, gp120, UFO-BG, and V1V2 scaffolds from A244 and other strains, using a Luminex multiple bead assay. We used a panel of mAbs with defined epitope specificities to verify the presence or absence of distinct V2 epitopes, V3 crown, and C-terminal C5 epitope on these antigens (**Figure S3**).

**Figure 4:**
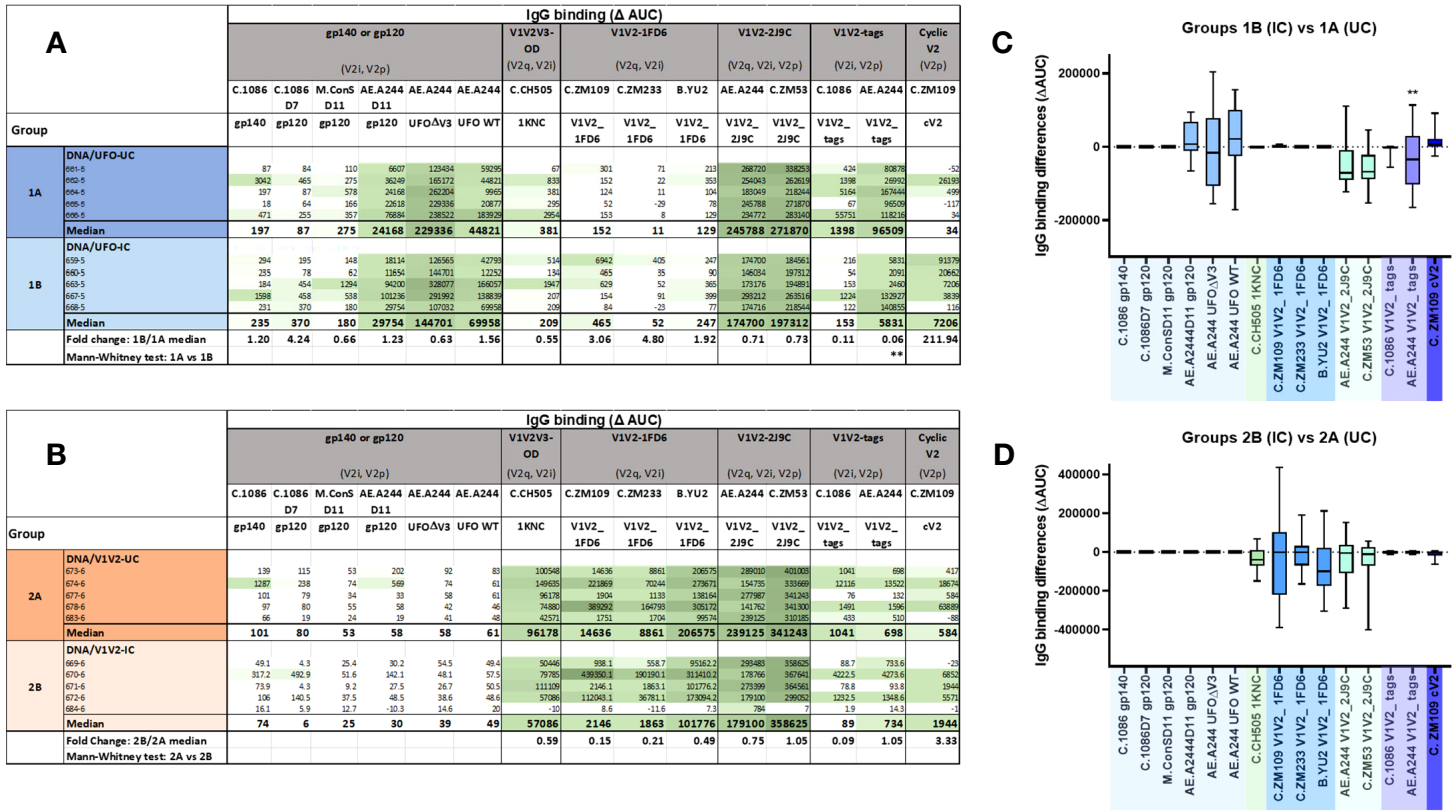
The profiles of binding Abs induced in immunized rabbits from the different groups. **(A, B)** Relative levels of Abs binding to 15 different antigens in Groups 1A, B **(A)** and Groups 2A, B **(B)**. Serially diluted serum samples from individual animals collected after the last vaccination were tested for IgG reactivity against 15 different antigens in the Luminex multiplex bead assays. Area under the titration curve (AUC) was calculated and subtracted by pre-bleed AUC to yield ΔAUC values. Median values and median fold changes of IC/UC were also calculated. **(C, D)** Differences in serum Ab levels elicited by ICs and their uncomplexed counterparts. AUC values from each of the five animals in Group 1B and Group 2B were subtracted by those of Group 1A and Group 2A, respectively. **p < 0.01; p > 0.05 was left unmarked.

Groups 1A and 1B (DNA/UFO-UC and IC) displayed a comparable pattern marked by robust recognition of all homologous A244 Env forms (gp120, UFO-BG.ΔV3, and UFO-BG.WT) as well as A244 V1V2 antigens (V1V2-2J9C and V1V2 tags) (**Figure 4A**). Varying degrees of cross-reactivity were also observed against heterologous C.ZM53 V1V2-2J9C and C.1098 V1V2 tags, whereas Ab reactivity with other V1V2 strains on 1FD6 or 1KNC scaffolds was weak or absent, indicating a limited breadth of anti-V1V2 Abs generated in Groups 1A and 1B.

Groups 2A and 2B (DNA/V1V2-UC and IC) exhibited a starkly different pattern of Ab reactivity from Groups 1A and 1B. Both groups 2A and 2B had strong Ab reactivity with the homologous V1V2-2J9C and cross-reactivity with V1V2 of clades B and C strains on different scaffolds (C.CH505 1KNC, C.ZM109 V1V2-1FD6, C.ZM233 V1V2-1FD6, B.YU2 V1V2-1FD6, and C.ZM53 V1V2-2J9C) (**Figure 4B**). However, the reactivity levels with V1V2 tags were low, and no reactivity was detected against gp120, gp140, or UFO-BG Env forms, including those of the A244 strain.

### Minimal changes in binding profiles of Abs induced by uncomplexed vs. complexed UFO-BG.ΔV3 and V1V2-2J9C

Comparisons between Groups 1A and 1B (DNA/UFO-UC vs. IC) revealed that Group 1B had a lower Ab response to V1V2 tags of A244, which contains V2i and V2p epitopes, whereas the responses to other antigens were not significantly different (**Figures 4A, C**). The response to C.ZM109 cV2 appeared to be higher in Group 1B vs. Group 1A. This pattern was also seen with other cV2 peptides, but a significant increase was only attained against A.MG505 cV2 (**Figure S4A**). These data indicate that the UFO-BG.ΔV3 IC administered to Group 1B caused a slight skewing of Ab responses toward the V2p type epitope and away from the V2i epitope recognized by 2158. However, no increase was apparent in reactivity against V1V2 scaffolds (1KNC, 1FD6, and 2J9C) bearing the V2 apex epitopes. Correlation analysis further showed weak or no positive correlation between Ab levels to Env (gp140 or gp120) and the different V1V2 antigens (**Figure S4B**).

A comparison of Group 2B vs. Group 2A (DNA/V1V2-IC vs. UC) also showed no augmentation of the Ab responses against V1V2 scaffolds bearing the V2 apex epitopes (**Figures 4B, D**). The median values against these V1V2 antigens tended to be lower in Group 2B vs. Group 2A. Ab responses against V1V2 tags or cV2 peptides also did not change substantially.

### Induction of PG9- and 2158-blocking Abs by uncomplexed vs. complexed UFO-BG.ΔV3 and V1V2-2J9C

A mAb blocking assay was subsequently used to delineate the presence of serum Abs that could compete with PG9 binding to the V2q epitope. No difference was detected in the levels of PG9 blocking by sera from Group 1B vs. Group 1A (DNA/UFO-IC vs. UC) (**Figure S5A**). A trend of higher PG9 blocking was observed with sera from Group 2B vs. Group 2A (DNA/V1V2-IC vs. UC), although a significant difference was not attained. These data indicate that the elicitation of PG9-like Abs was not improved by immunization with ICs of UFO-BG.ΔV3 and V1V2-2J9C as compared with their uncomplexed counterparts.

Immunization with 2158-containing ICs was expected to impede the induction of Abs against the V2i epitope recognized by 2158. In the blocking assay, we detected no to low levels of Abs capable of blocking 2158 binding to C.ZM109 V1V2 on the 1FD6 scaffold in all four groups of rabbits (**Figure S5B**). To examine whether the V2i Abs induced in these rabbits might be strain-specific, we tested sera to block 2158 binding to the homologous A244 V1V2 on 2J9C and found that 2158-blocking Abs were present in sera from all four groups (**Figure S5C**). The blocking levels were comparable, although they tended to be lower in Group 1B (DNA/UFO-IC). Therefore, IC vaccines containing 2158 still elicited robust Ab responses against the strain-specific V2i epitope, similar to the uncomplexed counterparts, indicating ineffective shielding of the V2i epitopes on these IC vaccines.

### Detection of virus neutralization in rabbits immunized with UFO-BG.ΔV3 but not with V1V2-2J9C

**Figure 5:**
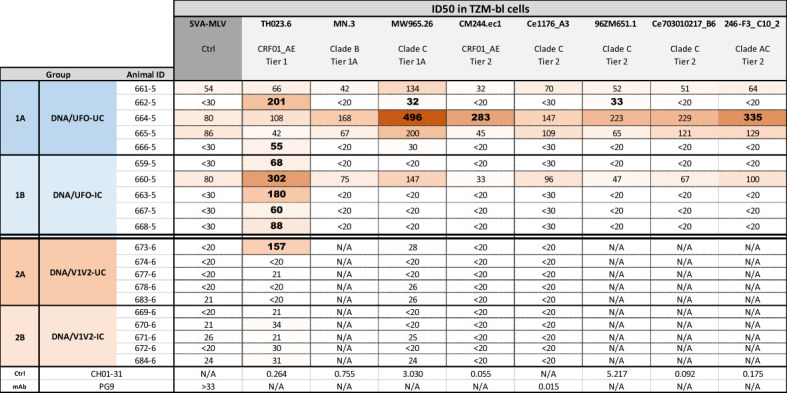
Neutralization activities elicited in the four rabbit groups. Neutralization of HIV-I pseudoviruses from different tiers and clades was tested in a standard assay using the TZM.bl target cells. Serum samples collected two weeks after the last immunization (week 14) were evaluated for neutralization activity. Virus pseudotyped with MLV was used as negative control, and control mAbs were also included for comparison. Bold values denote ID50 titers measurable above cut-offs (above MLV control <30 or >3-fold higher than MLV control of >30).

Serum neutralizing activity was assessed against HIV-1 pseudoviruses with tier 1 and tier 2 Env from clades B, C, AC, and AE, including TH023.6 and CM244.ec1 that are closely related to A244. A fraction of animals in Group 1A (DNA/UFO-UC) and all in Group 1B (DNA/UFO-IC) showed neutralization against tier 1 CRF01_AE TH023.6 (**Figure 5**). Group 1A (DNA/UFO-UC) also exhibited weak neutralization against few heterologous tier 1 and tier 2 viruses: Two rabbits (662-5 and 664-5) showed ID_50_ titers above the MLV control cutoff. In Group 1B (DNA/UFO-IC), neutralization against heterologous viruses was comparable to the MLV control. These data suggest that the IC vaccine with 2158 mAb did not favor the elicitation of a heterologous neutralization response.

In contrast, in Groups 2A and 2B (DNA/V1V2-UC and IC), only one rabbit displayed an ID_50_ of 157 against TH023.6. ID_50_ titers of the other rabbits against all viruses tested were below the MLV control cutoff. These results indicate that the anti-V1V2 Ab responses elicited in these groups were not accompanied with virus-neutralizing activity.

**Figure 6:**
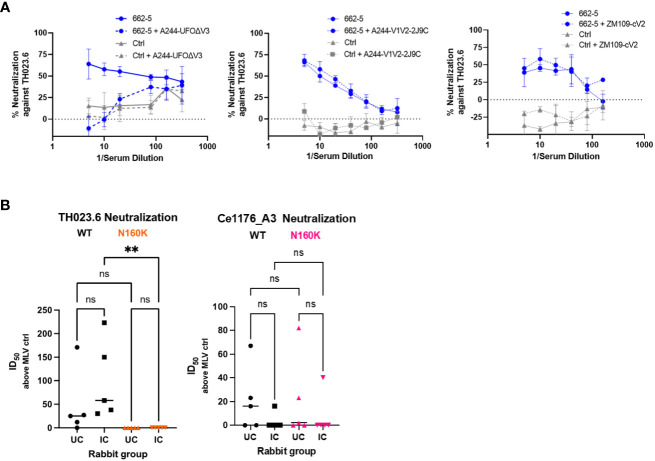
Diminishing TH023.6 neutralization by serum treatment with UFO-BG.ΔV3 or by N160K mutation. Virus neutralization was performed as in **Figure 5**. **(A)** Serially diluted serum was pre-incubated with UFO-BG.ΔV3, A244 V1V2-2J9C, or ZM109-cV2 peptide (each at a fixed amount of 50 µg/ml) before testing in the neutralization assay against TH023.6 virus. **(B)** Neutralizing activity of rabbit sera from Groups 1A and 1B against TH023.6 and Ce1176_A3 WT vs. the respective N160K mutants. ID_50_ titers above the MLV ID_50_ values are plotted.

To define the antigenic specificity of neutralizing Abs effective against TH023.6, serum 662-5, which had the highest ID_50_ titer against this virus, was pre-incubated with UFO-BG.ΔV3, V1V2-2J9C, or cV2 peptide prior to testing in a neutralization assay. The data in **Figure 6A** showed that TH023.6 neutralization was diminished by treatment with UFO-BG.ΔV3, but not with V1V2-2J9C or cyclic V2 antigens.

We further evaluated whether neutralization was dependent on N160 and its glycan, which are critical for recognition by PG9 and other V2 apex bNAbs (22, 23, 42). The N160K mutation abrogated TH023.6 neutralization detected in both Groups 1A and 1B with a significant reduction noted for Group 1B (**Figure 6B**). The same mutation was also tested in the context of Ce1176_A3, but positive neutralization against this virus was not detected and the N160K mutation had no significant effect. In line with its location in the V2 apex, N160K specifically abrogated neutralization of V2 apex-targeting bNAbs PG9, PG16, and PGDM1400 and did not reduce neutralizing potency of bNAbs against other sites, such as the CD4bs, V3 glycan, or membrane-proximal external region (MPER) (**Figure S6**). Of note, TH023.6 was resistant to V3 glycan bNAbs tested (PGT128, PGT121, and 10-1074), and the N160K mutation rendered TH023.6 sensitive to PGT128 but not PGT121 or 10-1074. This mutation also did not promote neutralization of the V3 crown– and V2i-specific mAbs, indicating that N160K did not expose these cryptic epitopes on Env. These data demonstrate that TH023.6-neutralizing Abs elicited in Groups 1A and 1B were dependent on the presence of N160 in the V2 apex, whereas neutralization against the other viruses were directed to yet undefined epitopes.

### Elicitation of V1V2-specific Abs capable of mediating ADCP

**Figure 7:**
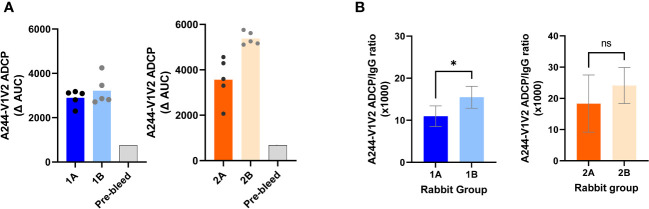
V1V2-specific ADCP activities detected in sera from the four rabbit groups. **(A)** Serially diluted serum was tested for ADCP with A244 V1V2-2J9C–coated beads and THP-1 phagocytes. Sera from immunized rabbits were tested in parallel with a pre-bleed serum pool. Titration curves were plotted for ADCP scores over serum dilutions. AUC values were then calculated and background-subtracted to yield ΔAUC. **(B)** ADCP potency for each rabbit serum was calculated as the ratios of ADCP/IgG against A244-V1V2-2J9C. *p < 0.05; ns, p > 0.05 by Mann–Whitney test. ns, not significant.

In addition to virus neutralization, Abs also mediate Fc-dependent effector functions that have been implicated in protection against HIV-1 (43–45). ADCP activities of rabbit Abs have been measured in a THP-1 phagocyte assay with antigen-coated fluorescent beads (33). Using the same assay, we examined V1V2-specific ADCP activities in sera from all four rabbit groups. The data show that all animals in the four groups had high levels of ADCP against V1V2-2J9C of A244 (**Figure 7A**). To compare the potency of Abs elicited by uncomplexed vs. complexed immunogens in Groups 1A and 1B and account for differences in the serum Ab levels, the ratios of ADCP AUC scores over IgG AUC levels against the same V1V2-2J9C antigen were calculated (**Figure 7B**). Group 1B showed a higher ADCP potency than Group 1A, indicating a qualitative difference in the V1V2-specific Abs elicited by the UFO-BG.ΔV3 IC as compared to that in the uncomplexed counterpart.

Comparison of Groups 2A and 2B showed slightly higher serum ADCP levels in Group 2B, which received the V1V2-2J9C IC (**Figure 7A**). However, the ratios of ADCP/IgG demonstrated comparably high ADCP potencies in both groups (**Figure 7B**). Hence, both complexed and uncomplexed V1V2-2J9C induce anti-V1V2 Abs with potent effector functions.

## Discussion

This study demonstrates the elicitation of neutralizing activity in sera from rabbits that received co-immunization of a V1V2-2J9C-encoding DNA vaccine and a UFO-BG.ΔV3 Env protein (Group 1A), whereas co-immunization with the same DNA vaccine and a V1V2-2J9C protein (Group 2A) was not effective. All vaccines presented the same CRF01_AE A244 sequence without the immunodominant V3 crown region. One notable rabbit in Group 1A showed heterologous neutralization against tier 1 (clade B) and tier 2 (clade AC and CRF01_AE) viruses, and another rabbit displayed homologous tier 1 neutralization (CRF01_AE) plus weak heterologous neutralization against tier 1 (clade B) and tier 2 (clade C). Hence, neutralization was attained sporadically in a fraction of animals, and the titers (ID_50_ < 500) were low. Nonetheless, these data indicate the capacity of vaccines based on a single CRF01_AE A244 Env sequence to elicit neutralizing Abs across multiple clades.

Epitope mapping using N160K variants revealed that the recognition of the N160-linked glycan at the V1V2 apex, a key element common to epitopes of PG9 and other V2-apex bNAbs (22, 23), was indispensable for tier 1 CRF01_AE TH023.6 neutralization, which indicates the targeting of the V2 apex by the vaccine-induced neutralizing Abs. A similar finding was reported when rhesus macaques were immunized with the same V1V2-2J9C DNA vaccine along with CM244 gp145 DNA and CM244 gp120 protein vaccines (27). Unlike the V2-apex bNAbs, the Abs elicited against this N160-dependent site were strain-specific; this was reminiscent of the V2-apex mAb 2909 that specifically neutralizes a tier 1 subtype B SF162 strain (42). Neutralization of heterologous viruses was mediated by Abs against yet undefined epitopes. These data suggest that co-immunization with V1V2-2J9C DNA and the UFO-BG.ΔV3 protein elicited Ab responses that conferred modest neutralization against tier 1 and limited tier 2 viruses from heterologous clades. Whether the UFO-BG V3 truncation contributes to heterologous neutralization by skewing Ab responses away from the V3 crown remains unclear and is under investigation. It should be noted, however, when a clade C C.1086 UFO design was constructed with mutations in the V2 and other Env regions without V3 crown removal, immunization of rabbits resulted in high titers of autologous neutralization especially against MW965.26 (46), a tier 1 clade C virus extremely sensitive to Abs against V3 crown and other cryptic epitopes (47, 48).

Immunization with an IC vaccine made of UFO-BG.ΔV3 and V2i mAb 2158 (Group 1B), which allosterically augments PG9 binding *in vitro*, resulted in a slight increase in the V2-apex-targeted neutralization of TH023.6. V1V2-specific ADCP potency was also higher in the DNA/UFO-IC group (Group 1B) as compared to that in the DNA/UFO-UC counterpart (Group 1A), although the improvement was modest and the samples size (5 animals per group) was small to allow for a robust statistical analysis. Nonetheless, this is in contrast with our past mouse experiments in which an A244 gp120 + 2158 IC vaccine generated no detectable tier 1 or tier 2 neutralization and did not improve ADCP against V1V2. An IC vaccine of JRFL gp120 and 2158, on the other hand, augmented neutralization against homologous clade B tier 1 virus mediated primarily by anti-V3 crown Abs (15). Neutralization against heterologous viruses, although only marginally detected in the DNA/UFO-UC group (Group 1A), was not observed in the DNA/UFO-IC group (Group 1B). The V2i mAb 2158 might pose steric hindrance and thus did not favor elicitation of neutralizing Abs against the heterologous viruses, but further investigation is necessary to test this idea and define the neutralizing epitopes effective against these viruses. The IC vaccine also may require further optimization. Although the UFO IC was prepared with a relatively high-affinity mAb (half max of 2158 for UFO: 0.62 nM) at a trimeric UFO/mAb ratio of 1:3 and thus expected to have each 2158 epitope fully occupied, Abs competing with 2158 were still generated in the DNA/UFO-IC group (Group 1B), indicating an incomplete or unstable IC formation. Moreover, because the IC vaccine was made with mAb 2158 of human IgG1, robust Ab responses were likely elicited against 2158 in the immunized rabbits, as observed in IC-immunized mice in our past study (49). Whether the anti-2158 Ab responses influence the induction of neutralizing antibodies against heterologous viruses are unknown and remain to be determined.

In contrast to neutralization, the antigen-binding IgG activities elicited by the DNA/UFO vaccines (Group 1A) displayed a more restricted breadth, mainly recognizing Env and V1V2 antigens of the homologous A244 strain. Group 1B immunized with UFO-BG.ΔV3 IC also produced a similar pattern, although varying increases in Ab levels against V2 peptides were observed in line with our past study with the A244 gp120 + 2158 IC vaccine (15). However, no significant correlation was apparent between Abs levels against V1V2 and Env antigens. Moreover, neutralization activity against the V2 apex was absorbed by UFO-BG.ΔV3, but not by V1V2-2J9C or V2 peptide. These results indicate that the neutralizing epitopes recognized by the vaccine-elicited Abs are not presented effectively by V1V2 antigens in our panel.

Co-immunization with V1V2-2J9C DNA and V1V2-2J9C protein vaccines (Group 2A) was intended to elicit Ab responses directed solely to V1V2. Although high levels of cross-reactive V1V2-specific Abs were elicited, the Abs did not recognize gp120, gp140, or UFO-BG Env proteins and had no neutralizing activity against the tier 1 or tier 2 viruses tested. The use of its IC counterpart (Group 2B) did not alter the binding and neutralization profiles. The lack of Env recognition and virus neutralization indicates that the elicited Ab responses may be directed to V1V2 conformations not present in the native Env structures. The V1V2-2J9C immunogens, however, were effective at directing Ab responses to V1V2 when used in a prime-boost regimen with gp120 or gp145 vaccines (27, 33, 36) or in co-immunization with UFO-BG.ΔV3 Env studied herein. A number of other V2 apex-targeting strategies have also been designed and tested for eliciting Ab responses targeted to the V2 apex. These strategies were exemplified by clade C-based UFO designs with structure-guided mutations at the V2 apex that elicited autologous neutralization in rabbits (46), germline targeting immunogens that activated the human V2-apex bNAb heavy-chain precursor-expressing B cells in knock-in mice (26), V2-apex bNAb inferred precursor-binding SOSIPs that improved exposure of the V2-apex region (49), and signature-based epitope targeted vaccines that broadened neutralizing Ab responses in immunized guinea pigs (50). Nonetheless, vaccines capable of generating the prototypic V2-apex bNAbs in wild-type animals remain elusive.

This study demonstrates that co-immunization of rabbits with a V1V2-targeting DNA vaccine and UFO-BG Env immunogen lacking the immunodominant V3 crown resulted in elicitation of neutralizing Abs against some heterologous tier 1 and limited tier 2 viruses. The neutralization breadth was manifested by Abs against V2 apex and possibly other undefined sites. The data further point to the potential interference of pre-existing Abs and ICs early during infection in eliciting neutralizing Abs.

## Data availability statement

The original contributions presented in the study are included in the article/**Supplementary Material**. Further inquiries can be directed to the corresponding author.

## Ethics statement

The animal study was approved by The Icahn School of Medicine at Mount Sinai Institutional Animal Care and Use Committee. The study was conducted in accordance with the local legislation and institutional requirements.

## Author contributions

CEH: Conceptualization, Formal Analysis, Funding acquisition, Resources, Supervision, Writing – original draft, Writing – review & editing. XL: Data curation, Formal Analysis, Investigation, Methodology, Resources, Validation, Writing – review & editing. AB: Data curation, Formal Analysis, Investigation, Writing – review & editing. DH: Data curation, Formal Analysis, Investigation, Methodology, Writing – review & editing. JK: Formal Analysis, Methodology, Visualization, Writing – review & editing. PR: Data curation, Investigation, Writing – review & editing. CL: Investigation, Resources, Writing – review & editing. XJ: Investigation, Methodology, Resources, Writing – review & editing. SP: Formal Analysis, Investigation, Methodology, Resources, Writing – review & editing. TO: Formal Analysis, Investigation, Methodology, Resources, Writing – review & editing. PB: Formal Analysis, Investigation, Methodology, Resources, Writing – review & editing. MT: Formal Analysis, Investigation, Methodology, Resources, Software, Writing – review & editing. JZ: Methodology, Resources, Writing – review & editing. AN: Formal Analysis, Software, Writing – review & editing. SZ-P: Resources, Writing – review & editing. CU: Data curation, Formal Analysis, Funding acquisition, Supervision, Writing – review & editing. XS: Data curation, Formal Analysis, Funding acquisition, Investigation, Methodology, Resources, Writing – review & editing. X-PK: Funding acquisition, Project administration, Resources, Supervision, Writing – review & editing. AH: Conceptualization, Funding acquisition, Resources, Supervision, Writing – review & editing.
