# Corrigendum: Vaccination with immune complexes modulates the elicitation of functional antibodies against HIV-1

**DOI:** 10.3389/fimmu.2023.1329069

**Published:** 2023-11-09

**Authors:** Catarina E. Hioe, Xiaomei Liu, Andrew N. Banin, Daniel W. Heindel, Je´romine Klingler, Priyanka G. Rao, Christina C. Luo, Xunqing Jiang, Shilpi Pandey, Tracy Ordonez, Philip Barnette, Maxim Totrov, Jiang Zhu, Arthur Na´das, Susan Zolla-Pazner, Chitra Upadhyay, Xiaoying Shen, Xiang-Peng Kong, Ann J. Hessell

**Affiliations:** ^1^Division of Infectious Diseases, Department of Medicine, Icahn School of Medicine at Mount Sinai, New York, NY, United States; ^2^Research Service, James J. Peters VA Medical Center, Bronx, NY, United States; ^3^Department of Biochemistry and Molecular Pharmacology, New York University Grossman School of Medicine, New York, NY, United States; ^4^Division of Pathobiology and Immunology, Oregon National Primate Research Center, Oregon Health and Science University, Beaverton, OR, United States; ^5^Molsoft L.L.C., San Diego, CA, United States; ^6^Department of Integrative Structural and Computational Biology and Department of Immunology and Microbiology, The Scripps Research Institute, La Jolla, CA, United States; ^7^Department of Environment Medicine, New York University Grossman School of Medicine, New York, NY, United States; ^8^Division of Surgical Sciences, Department of Surgery, Duke University School of Medicine, Durham, NC, United States

**Keywords:** HIV-1 vaccine, HIV-1 Env, antibody, immune complex (IC), virus neutralization, ADCP

In the published article, there was an error in **Figure 5** as published. MW965.26 was incorrectly listed as Clade B. It should be Clade C. The corrected **Figure 5** and its caption appear below.

**Figure 5:**
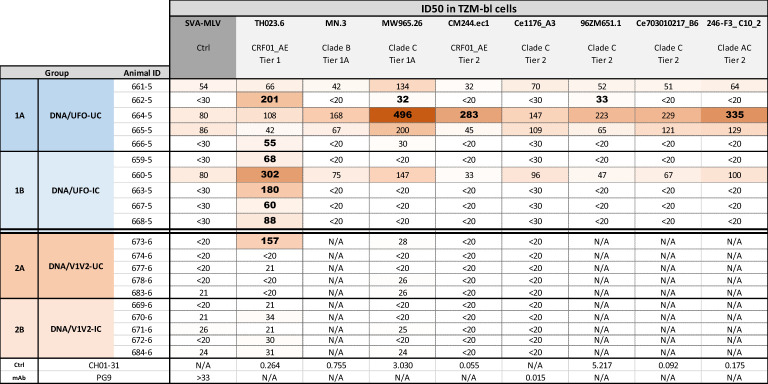
Neutralization activities elicited in the four rabbit groups. Neutralization of HIV-I pseudoviruses from different tiers and clades was tested in a standard assay using the TZM.bl target cells. Serum samples collected two weeks after the last immunization (week 14) were evaluated for neutralization activity. Virus pseudotyped with MLV was used as negative control, and control mAbs were also included for comparison. Bold values denote ID_50_ titers measurable above cut-offs (above MLV control <30 or >3-fold higher than MLV control of >30).

The authors apologize for this error and state that this does not change the scientific conclusions of the article in any way. The original article has been updated.

