# Supplementary material for: Vaccination with immune complexes modulates the elicitation of functional antibodies against HIV-1
Source: Front Immunol. 2023 Oct 3;14:1271686. doi: 10.3389/fimmu.2023.1271686 (PMC10579950; doi:10.3389/fimmu.2023.1271686)
Supplement: Supplementary file 1 [file DataSheet_1.pdf]

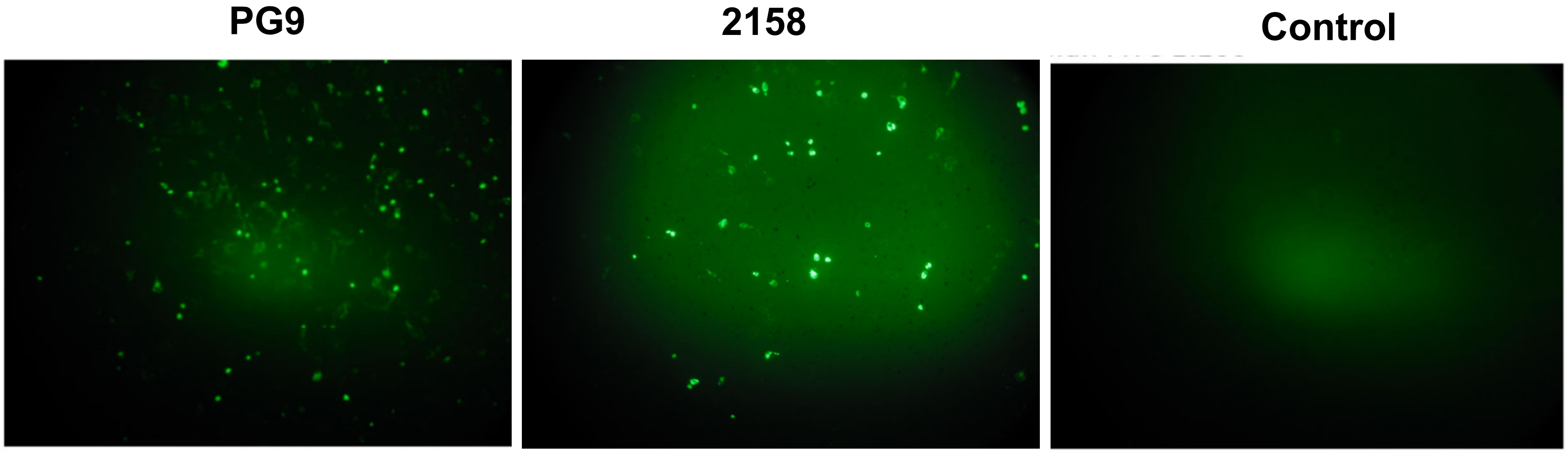

Figure S1. Antigenicity profile of V1V2-2J9C. A DNA plasmid 418H encoding A244 V1V2-2J9C was coated on 1 µm-gold beads and transfected using a gene gun into COS-7 cells. Protein expression and antibody reactivity of V1V2-2J9C were evaluated using V2q mAb PG9 and V2i mAb 2158 (2 µg/ml each), followed by FITC-labeled goat anti-human IgG. Untreated COS-7 cells stained with antibodies served as control.

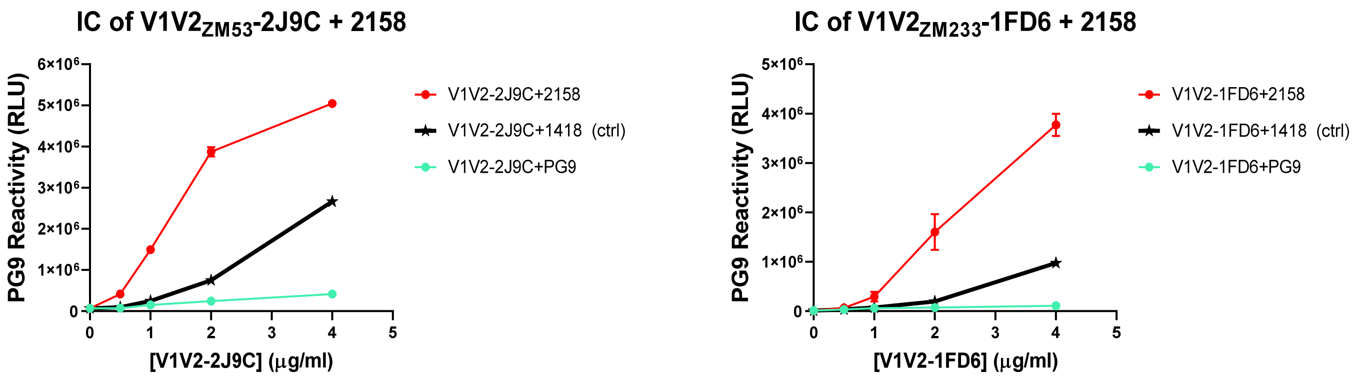

Figure S2. Enhanced PG9 reactivity with different V1V2-scaffolds in complex with 2158.
ZM53 V1V2-2J9C or ZM233 V1V2-1FD6 were pre-incubated with 2158, PG9, or 1418 (irrelevant control) at a 1:3 ratio. The antigen-mAb mixtures were diluted serially, coated onto wells, and reacted with biotinylated PG9, followed with AP-streptavidin and luminescent substrate.

		gp140 or gp120						V1V2V3-	V1V2-1FD6			V1V2-2J9C		V1V2-tags		Cyclic V2
**Epitope**	**mAb**	AE.A244-UFO ΔV3	AE.A244-UFO-WT	AE.A244 D11 gp120	C.1086 gp140	C. 1086 D7 gp120	M.Con-s D11 gp120	C.CH505-1KNC	C.ZM233 V1V2-1FD6	C.ZM109 V1V2-1FD6	B.YU2 V1V2-1FD6	V1V2/A244-2J9C	V1V2/ZM53-2J9C	AE.A244 V1V2.tags	C.1086 V1V2.tags	cV2/C.ZM109
V2q	PG9	2.26	2.26	0.36	0.22	0.12	0.34	2.76	1.87	2.63	2.36	3.14	2.44	0.26	0.35	0.23
V2i	2158	4.34	4.33	2.35	2.18	2.15	1.88	0.30	4.38	4.51	4.60	4.55	4.48	2.14	1.72	0.20
V2p	CH58	4.51	4.46	2.44	2.27	2.39	2.08	0.24	0.18	0.31	0.27	4.52	4.03	2.42	2.40	0.11
	CH59	4.51	4.52	2.38	2.42	2.32	0.24	0.17	0.24	0.64	0.16	4.51	0.17	2.47	2.49	2.37
	CAP228.3D	4.48	4.57	2.41	2.36	2.42	2.06	0.18	0.20	0.21	0.27	4.48	4.49	2.48	2.44	0.12
	CAP228.16	4.50	4.53	2.10	2.39	2.23	2.06	0.17	0.20	0.19	0.43	4.39	4.49	2.48	2.45	0.07
V3	2558	0.18	4.56	2.29	2.45	2.41	2.09	3.06	0.25	0.20	0.19	0.20	0.18	0.09	0.13	0.07
	447	0.23	0.43	0.40	2.42	2.22	1.96	0.69	0.46	0.28	0.42	0.24	0.22	0.10	0.29	0.09
C5	670	0.21	0.31	0.66	0.51	0.32	2.21	0.24	0.23	0.30	0.20	0.22	0.27	0.35	0.24	0.32
Ctrl	860	0.14	0.14	0.12	0.08	0.08	0.11	0.15	0.16	0.17	0.15	0.14	0.15	0.09	0.09	0.16
	PBS	0.12	0.13	0.12	0.07	0.06	0.14	0.12	0.12	0.13	0.13	0.13	0.12	0.06	0.07	0.07

Figure S3. Recognition of 15 antigens by anti-Env mAbs with defined epitope specificities.

ELISA was performed to test antigen reactivity with mAbs specific for three distinct V1V2 epitopes (V2q or V2 apex, V2i, and V2p), the V3 crown epitopes, the C terminus (C5) of gp120, and an irrelevant parvovirus antigen (860). Based on the OD values above control, the presence of V2q, V2i, and/or V2p epitopes in each of the 15 antigens is denoted in Figures 4A and 4B.

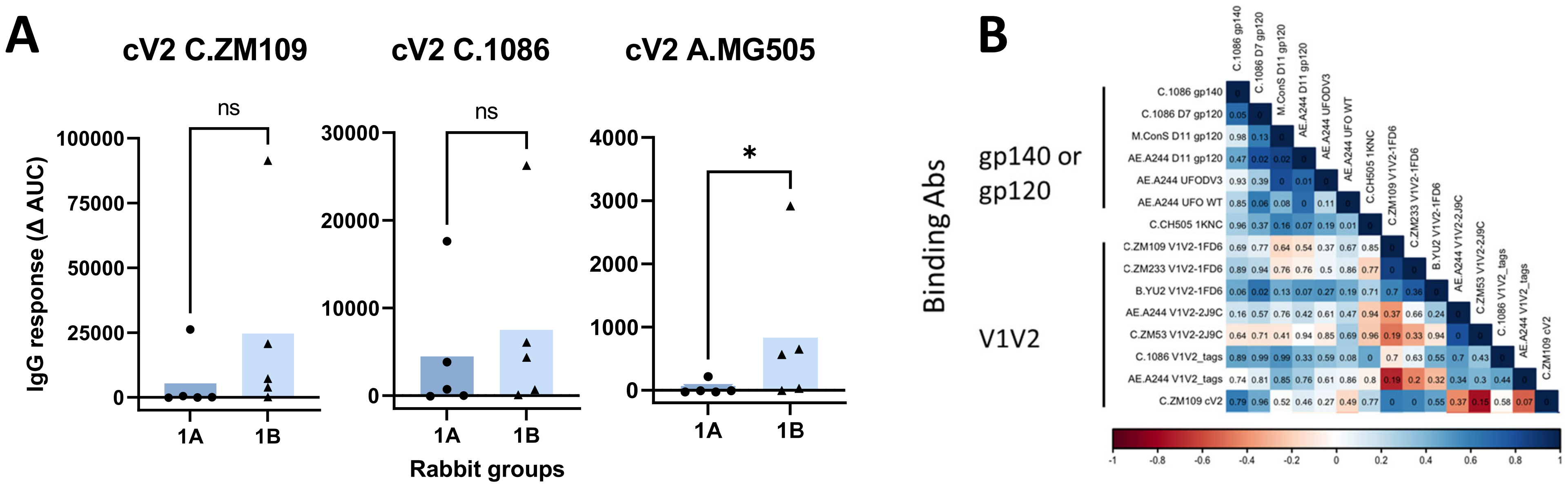

Figure S4.

A. Comparison of Ab responses to cyclic V2 (cV2) peptides elicited in Groups 1A vs 1B.
Beads coated with each cV2 peptide were reacted with serially diluted sera after the last vaccination dose. AUCs were subtracted with pre-bleed AUCs to yield ΔAUC values. *, $p<0.05$; ns, $p>0.05$ by Mann Whitney test.

B. Correlation of IgG binding data from the multiplex bead assays for Groups 1A and 1B. Multiple bead assay data were from Figure 4. Correlation analysis was performed using the Spearman test. Correlation coefficients are color coded according to the red (-1)-to-blue (+1) scale, while p values were presented in each box.

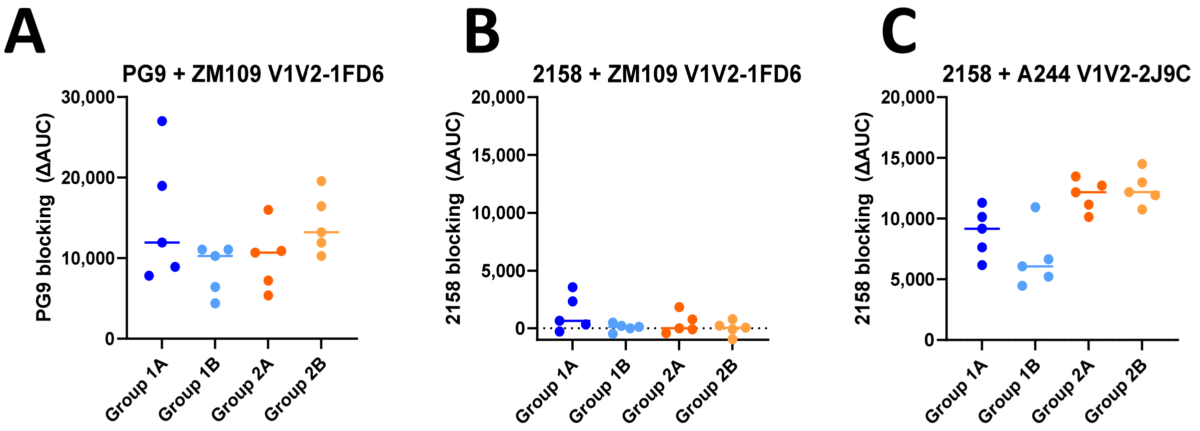

Figure S5. Elicitation of Abs capable of blocking PG9 and 2158 in the four rabbit groups. Beads coated with C.ZM109 V1V2-1FD6 or A244 V1V2-2J9C (both bearing V2q and V2i epitopes) were treated with serially diluted immune sera collected after the final vaccination or pre-bleed serum pool. Biotinylated PG9 or 2158 mAbs were subsequently added, and the reduced binding of the respective mAbs by immune sera was presented as ΔAUC (AUC of prebleed sera - AUC of immune sera).

A. Relative levels of PG9-blocking Abs in immune sera binding to ZM109 V1V2-1FD6.

B. Lack of 2158-blocking Abs in immune sera when tested with ZM109 V1V2-1FD6.

C. Relative levels of 2158-blocking Abs in immune sera when tested with A244 V1V2-2J9C.

mAb	Epitope	TH023.6	TH023.6 N160K
		IC50 (µg/ml)	
PG9	V2 apex	**0.11**	**>5**
PG16	(aka V2 glycan or V2q)	**3.13**	**>5**
PGDM1400		**0.007**	**>5**
CH01		>25	>25
PGT128	V3-glycan	>5	**0.09**
PGT121		>5	>5
10-1074		>25	>25
sCD4	CD4bs	**0.87**	**0.62**
b12		>25	>25
N6		**0.01**	**0.05**
VRC01		**1.09**	**0.24**
3BNC117		**0.05**	**0.06**
CH31		**0.2**	**0.36**
DH235_4A		**1.61**	**1.37**
CH103		>25	>25
PGT151	gp120/gp41	>10	>5
VRC34.01		>25	>5
2G12	Glycan	>25	>25
DH511.2_K3	MPER	**0.012**	**0.011**
2F5		<0.01	<0.01
4E10		<0.01	**0.03**
697-30D	V2i	>25	>25
3074	V3 crown	**0.03**	**1.87**
447-52D		>25	>25

Figure S6. N160K abrogated TH023.6 neutralization by V2-apex mAbs without affecting the other mAbs.
